# Personality organization and insight into psychosis in patients in the early stages of psychotic disorders and their parents

**DOI:** 10.1192/j.eurpsy.2026.11733

**Published:** 2026-07-31

**Authors:** L. Goršić, S. Caratan, S. Kocijan, M. Krevatin, H. Goršić, V. Grosic

**Affiliations:** 1 Day Hospital for non psychotic disorders, University Psychiatric Hospital Sveti Ivan, Zagreb; 2 Department of Psychiatry and Neurology, Faculty of Dental Medicine and Health J.J. Strossmayer, Osijek; 3 Acute Male Psychiatric Unit; 4 Integrative Psychiatry; 5 Department of Psychiatry and Neurology, University Psychiatric Hospital Sveti Ivan, Zagreb, Croatia

## Abstract

**Introduction:**

Personality development is shaped by genetic predisposition and early caregiver relations. Through the interplay of temperament and early experiences, internalized representations of self and others are formed. The degree of integration of good and bad representations, along with self–object differentiation, leads to three levels of personality organization: neurotic (NO), borderline (BO), and psychotic (PO). These differ in defense mechanisms (mature vs. immature), identity coherence, and reality testing. PO and BO are marked by immature defenses (splitting, projection, projective identification), greater identity diffusion, and impaired reality testing (though preserved in BO). NO is defined by stable identity, mature defenses, and intact reality testing. In early psychosis (≤5 years), impaired insight is common and linked to delayed treatment, poor adherence, aggression, and worse long-term outcomes. Clinical insight is a multidimensional construct involving awareness of illness, consequences, treatment compliance, and recognizing symptoms as pathological.

**Objectives:**

Aim of this study was to investigate level of patient’s and parent’s personality

organization with clinical insight into early psychosis.

**Methods:**

We conducted a cross-sectional study on 105 parent–child dyads with early-stage psychotic disorders (≤5 years; ICD-10 F20–F29), including patients aged 18–35 years of both sexes, hospitalized at *Sveti Ivan* Psychiatry Clinic. All participants gave informed consent. Instruments included a sociodemographic questionnaire, the abbreviated SUMD, PANSS (five-factor model), CGI-S, and Kernberg IPO. The study followed the Helsinki Declaration.

**Results:**

Patients were predominantly male, well educated, and most often diagnosed with F23; among parents, mothers were more represented. On the PANSS Five-Factor scale, patients improved across all domains. Patients showed greater illness awareness than parents, with fathers more aware than mothers. Compared to patients, parents demonstrated more mature defenses, less identity diffusion, and better reality testing. In parents, identity diffusion was linked to lower illness awareness and reduced recognition of social consequences, but not to insight into medication needs.

**Conclusions:**

Insight is a complex, dynamic phenomenon that can only be meaningfully assessed in those with lived experience of psychosis. This study highlights the intricate parent–child dynamics and underscores the need for family psychotherapy and psychosocial interventions in early psychosis treatment.

**Disclosure of Interest:**

None Declared

